# Machine learning-driven discovery of novel therapeutic targets in diabetic foot ulcers

**DOI:** 10.1186/s10020-024-00955-z

**Published:** 2024-11-14

**Authors:** Xin Yu, Zhuo Wu, Nan Zhang

**Affiliations:** 1 Pediatric Oncology of the First Hospital of Jilin University, Changchun, 130021 China; 2 Mircrosurgery Department of PLA General Hospital, Beijing, 100853 China; 3 Burn Department of the First Hospital of Jilin University, No. 1 Xinmin Street, Chaoyang District, Changchun, 130021 Jilin Province China

**Keywords:** Diabetic Foot Ulcers, Transcriptome sequencing, Machine learning, SCUBE1, RNF103-CHMP3, Early diagnosis

## Abstract

**Background:**

To utilize machine learning for identifying treatment response genes in diabetic foot ulcers (DFU).

**Methods:**

Transcriptome data from patients with DFU were collected and subjected to comprehensive analysis. Initially, differential expression analysis was conducted to identify genes with significant changes in expression levels between DFU patients and healthy controls. Following this, enrichment analyses were performed to uncover biological pathways and processes associated with these differentially expressed genes. Machine learning algorithms, including feature selection and classification techniques, were then applied to the data to pinpoint key genes that play crucial roles in the pathogenesis of DFU. An independent transcriptome dataset was used to validate the key genes identified in our study. Further analysis of single-cell datasets was conducted to investigate changes in key genes at the single-cell level.

**Results:**

Through this integrated approach, SCUBE1 and RNF103-CHMP3 were identified as key genes significantly associated with DFU. SCUBE1 was found to be involved in immune regulation, playing a role in the body’s response to inflammation and infection, which are common in DFU. RNF103-CHMP3 was linked to extracellular interactions, suggesting its involvement in cellular communication and tissue repair mechanisms essential for wound healing. The reliability of our analysis results was confirmed in the independent transcriptome dataset. Additionally, the expression of SCUBE1 and RNF103-CHMP3 was examined in single-cell transcriptome data, showing that these genes were significantly downregulated in the cured DFU patient group, particularly in NK cells and macrophages.

**Conclusion:**

The identification of SCUBE1 and RNF103-CHMP3 as potential biomarkers for DFU marks a significant step forward in understanding the molecular basis of the disease. These genes offer new directions for both diagnosis and treatment, with the potential for developing targeted therapies that could enhance patient outcomes. This study underscores the value of integrating computational methods with biological data to uncover novel insights into complex diseases like DFU. Future research should focus on validating these findings in larger cohorts and exploring the therapeutic potential of targeting SCUBE1 and RNF103-CHMP3 in clinical settings.

**Supplementary Information:**

The online version contains supplementary material available at 10.1186/s10020-024-00955-z.

## Highlights

1. This is the first report identifying SCUBE1 and RNF103-CHMP3 as therapeutic targets for diabetic foot ulcers (DFU).

2. Our study found that SCUBE1 and RNF103-CHMP3 were significantly downregulated in patients who were cured of DFU.

3. This study discovered that SCUBE1 and RNF103-CHMP3 are associated with extracellular interactions.

4. Our findings indicate that SCUBE1 and RNF103-CHMP3 are related to immune cell infiltration.

5. This study provides new theoretical foundations and molecular targets for the diagnosis and treatment of DFU.

**Supplementary Information:**

The online version contains supplementary material available at 10.1186/s10020-024-00955-z.

## Introduction

Diabetic foot ulcers (DFU) are common and serious complications among diabetic patients, characterized by high incidence and disability rates, which impose a heavy burden on patients and their families (Cloete 2022; McDermott et al. 2023; Sorber and Abularrage 2021). The pathogenesis of DFU is complex and multifactorial, involving various factors such as poor blood sugar control, peripheral neuropathy, peripheral arterial disease, and infection. These factors interact, leading to difficulties in wound healing in the patients’ feet (Bodman et al., 2024; Guo et al. 2023). Despite continuous advancements in modern medical technology, the treatment of DFU remains a significant challenge (Holl et al. 2021; Deng et al. 2023; Subramaniam et al. 2021). Globally, millions of patients undergo lower limb amputations annually due to DFU, severely affecting their quality of life of patients but also imposing a substantial economic burden on healthcare systems (Gong et al. 2023; Enweluzo et al. 2021; Collins et al. 2022). With the continuous increase in the prevalence of diabetes, the incidence of DFU continues to increase, highlighting the urgent need for in-depth research into its pathogenesis to develop more effective diagnostic tools and treatment strategies (OuYang et al. 2023; Kaka et al. 2023).

Currently, the clinical management of DFU primarily relies on traditional wound care, antimicrobial therapy, and blood sugar control (McDermott et al. 2023; Wang et al. 2022a; Yang et al. 2022). However, the incomplete understanding of the molecular mechanisms underlying DFU limits the efficacy of existing diagnostic tools and treatments, often resulting in high recurrence rates (Liu et al. 2023; Deng et al. 2023; Yu et al. 2023). Conventional treatment approaches often focus on infection control and improving local blood circulation but lack specificity towards addressing the fundamental mechanisms of the pathology (Deng et al. 2023; Barutta et al. 2022; Yang et al. 2023). This scenario highlights a significant bottleneck in the field of DFU research, namely the lack of a profound understanding of its molecular biology mechanisms (Zhang et al. 2023a; Zou et al. 2022; Miron et al. 2023). Therefore, exploring the molecular biology mechanisms of DFU, especially genes and pathways associated with its treatment response, holds crucial clinical significance (Zhang et al. 2023a; Zou et al. 2022; Miron et al. 2023). This endeavor facilitates the development of more personalized and precise treatment strategies, ultimately improving patient outcomes (Russo et al. 2023; Tran and Haley 2021; Qi et al. 2023).

Transcriptomic technology, which comprehensively analyzes gene expression patterns, has emerged as a powerful tool for uncovering disease-related genes and molecular pathways, making it indispensable for investigating the molecular mechanisms of complex diseases (Benck et al. 2022; Lu and Keleş 2023). Building on this foundation, the integration of machine learning techniques has introduced novel methods and tools for analyzing high-throughput data (Tran et al. 2021; Heumos et al. 2023; Ding et al. 2022). Machine learning algorithms excel at managing vast biological datasets, allowing for the identification of disease-associated genes and predicting their roles in disease diagnosis and treatment (Su et al. 2022; Dai et al. 2024; Song et al. 2023). By combining differential expression analysis with machine learning algorithms, key genes related to DFU can be identified from extensive transcriptome data, offering new perspectives for understanding its molecular mechanisms (Zhang et al. 2023b; Zhou et al. 2024). These genes not only serve as biomarkers for early diagnosis but also present potential targets for therapeutic interventions (Li et al. 2022; Yao et al. 2021; Feng et al. 2022).

This study utilized transcriptome data related to DFU downloaded from the Gene Expression Omnibus (GEO) database to conduct differential expression analysis, identify candidate genes, and conduct Gene Ontology (GO), Kyoto Encyclopedia of Genes and Genomes (KEGG), and Disease Ontology (DO) enrichment analyses to unveil the significant pathway alterations and biological processes in DFU patients after treatment. LASSO regression model and SVM-RFE machine learning algorithms were subsequently employed to further identify feature genes associated with DFU treatment response, evaluating their performance in DFU diagnosis through ROC curves and AUC values. Additionally, regulatory networks of core feature genes were explored, and in combination with the single-sample gene set enrichment analysis (ssGSEA) analysis algorithm, the impact of these feature genes on the molecular mechanisms of DFU was elucidated. The integration of these methods not only enhances the precision and reliability of the research but also provides new insights into unraveling the intricate molecular mechanisms underlying DFU.

This study aims to employ machine learning techniques to analyze transcriptome sequencing data, identifying genes associated with the treatment response of DFU to provide new molecular targets for the early diagnosis and treatment of DFU. By identifying feature genes related to the treatment response of DFU, such as SCUBE1 and RNF103-CHMP3, and exploring their association with immune cell regulation and inflammatory responses, we aim to offer novel strategies and directions for the prevention and treatment of DFU in the future. Specifically, SCUBE1 and RNF103-CHMP3 exhibit significant expression changes in DFU patients and are closely linked to immune responses and extracellular interactions. These gene alterations may directly impact the regulation of inflammatory responses and wound-healing processes. Therefore, the findings of this study not only contribute to enhancing the accuracy of DFU diagnosis but also have the potential to facilitate the implementation of personalized therapies to improve clinical outcomes for patients. These research findings provide essential molecular targets and new strategic directions for the prevention and treatment of DFU in the future.

## Materials and methods

### Data download

Transcriptome data set GSE230426 related to DFU was downloaded from the GEO database (https://www.ncbi.nlm.nih.gov/geo/). Fifteen samples were selected from patients with DFU infection at 0 weeks after treatment as the control group data set, while 15 samples from patients who had recovered at 8 weeks after infection were selected as the experimental group data set for a series of bioinformatics analyses.

The feature genes identified through machine learning were validated using the GSE80178 dataset from the GEO database. For this purpose, three diabetic non-foot ulcer samples and six diabetic foot ulcer samples were selected to further evaluate the expression differences of these genes in different conditions. Additionally, PBMC samples from the GSE165816 dataset in the GEO database, which included three subjects with cured DFU and two subjects with uncured DFU, were downloaded.

### Differential analysis

Differential gene expression analysis was performed on the sequencing data, comparing the samples from the control group at the start of treatment (0 weeks) with those from the experimental group at 8 weeks post-treatment using the “limma” package in R software. Genes with │logFC│>1 and *P*-value < 0.05 were considered differentially expressed. The results of the differential analysis were visualized using the “ggplot2” package in R software to plot volcano plots.

The “VennDiagram” package in R software was used to identify common genes and create a Venn diagram to visualize the intersection of relevant genes.

### Enrichment analysis

For gene set functional enrichment analysis, the gene-GO annotations from the org.Hs.eg.db package (version 3.1.0) in R software were utilized as the background. The genes were mapped to the background set, and the enrichment analysis was carried out using the clusterProfiler package (version 3.14.3) in R. A minimum gene set size of 5 and a maximum gene set size of 5000 were designated for the analysis. The GO analysis comprised biological processes (BP), molecular functions (MF), and cellular components (CC) analyses to detect enriched pathways. The objective of this analysis was to reveal the cellular functions, signaling pathways mainly influenced by candidate target genes, and enriched pathways associated with disease-related differential genes. For the DO enrichment analysis, the R package org.Hs.eg.db (version 3.1.0) was employed to access gene annotations for the gene set, offering crucial background information. Subsequently, these genes were linked to the background set of DO to guarantee the association of each gene with a disease classification in the DO system. In the gene set functional enrichment analysis, the latest gene annotations from the KEGG Pathway were obtained using the KEGG REST API as the background. The genes were then connected to the background set, and the enrichment analysis was conducted utilizing the R package clusterProfiler (version 3.14.3) to assess the enrichment of the gene set. A minimum of 5 genes and a maximum of 5000 genes were specified, with a *P*-value of < 0.05 considered as a significant criterion for enrichment.

### Least absolute shrinkage and selection operator (LASSO) regression algorithm

In our bioinformatics research, the LASSO regression was employed to identify key genes associated with diseases. To ensure experiment reproducibility, a random seed was initially set, and the glmnet package was utilized to handle datasets containing a large number of variables. The candidate differentially expressed genes (DEGs) underwent preprocessing and were subjected to LASSO regression using the glmnet function. The data was treated as a binary classification problem, with the response variable derived from sample names using regular expressions. Model evaluation was performed by plotting the model object and employing cross-validation with cv.glmnet to determine the optimal lambda value. Ultimately, genes corresponding to non-zero coefficients obtained using the optimal lambda value were regarded as key genes linked to disease status and were then outputted. This approach not only accurately selected key genes but also improved prediction accuracy by reducing model overfitting, thereby effectively supporting biomarker discovery and research into disease mechanisms.

### Support vector machine - recursive feature elimination (SVM-RFE) method

The SVM-RFE method was applied to identify key gene expression features associated with disease status. The analysis was initiated with the environment being set up by loading the necessary R packages “e1071,” “kernlab,” and “caret,” and followed by the retrieval of DEGs. Feature selection was carried out using the rfe function based on cross-validation, with SVM utilizing a radial basis kernel function and varying feature subset sizes. The evaluation of the analysis results was conducted by plotting the relationship between the number of features and the root mean square error (RMSE) of the model. Once the optimal feature set was determined, the relevant genes were outputted. This method effectively selects key gene expression features, providing a scientific basis for further research on diseases.

### Construction of protein-protein interaction (PPI) network

A network of regulatory interactions among genes was built, and their regulatory factors were predicted using the GeneMANIA database (http://www.string-db.org/).

### Immune cell correlation analysis

ssGSEA is a computational method used to assess the relative enrichment level of a specific gene set in an individual sample. This method can generate an enrichment score for each sample, indicating whether the expression activity of a specific gene set in that particular sample has been upregulated or downregulated relative to the background gene expression. The ssGSEA method ranks genes based on expression data and then calculates an enrichment score for each gene set using an empirical cumulative distribution function (ECDF) based on the ranking positions of genes within and outside the gene set. The score is calculated by comparing the relative positions of genes within the gene set to those outside the gene set in the ranking, thus obtaining a score that reflects the relative enrichment level of gene set expression.

The experimental procedure begins by importing the necessary R packages (including reshape2, ggpubr, limma, GSEABase, and GSVA) to set up the environment for data processing and analysis. Next, establish the working directory and read the preprocessed transcriptome data, which contains normalized gene expression values. Upon data import, convert it to a matrix format and ensure that the row names (gene names) and column names (sample names) are correctly set. Subsequently, utilize the getGmt() function to load the information defining the preselected immune-related gene sets from a .gmt file.

The ssGSEA analysis was executed using the gsva() function with the parameters method=’ssgsea’ to specify the use of the ssGSEA method, kcdf=’Gaussian’ to define the kernel density estimation function as a Gaussian distribution, and abs.ranking = TRUE to indicate the use of absolute ranking of genes for score computation. Upon completing the analysis, the ssGSEA scores obtained for each sample were normalized in preparation for subsequent statistical analysis and comparisons. Finally, the processed normalized ssGSEA scores were saved.

### Single-cell transcriptome data processing and analysis

In the single-cell RNA sequencing (scRNA-seq) analysis pipeline, the data were first normalized using the LogNormalize method with a normalization factor of 10,000 to ensure the comparability of gene expression levels across different cells. Next, the FindVariableFeatures method was used to select highly variable genes (top 2000), which helps better characterize cell-to-cell differences in subsequent analyses. To further remove batch effects, the Harmony algorithm was applied for batch correction, making the data from different experimental batches more comparable. Cell cycle scoring was then performed to correct for the potential influence of the cell cycle on the data.

Principal component analysis (PCA) was used for dimensionality reduction, and the JackStraw method was employed to select significant principal components, providing a foundation for subsequent clustering and visualization. For cell clustering, t-SNE, a nonlinear dimensionality reduction method, was used for cluster analysis, and the FindClusters function was utilized to group cells, with the clustering results optimized by adjusting different resolution parameters. The overall quality control workflow included steps such as normalization, batch correction, dimensionality reduction, and cell cycle correction to ensure the accuracy and reliability of the data analysis.

### Statistical software and data analysis methods

This research utilized R software version 4.2.1, compiled through the integrated development environment RStudio version 4.2.1. Perl language was employed for file processing, with Perl version 5.30.0. The network visualization tool Cytoscape was utilized (version 3.7.2), along with the statistical software SPSS (IBM SPSS Statistics, Chicago, IL, USA, version 21.0). Descriptive statistics were presented as means ± standard deviations, and between-group comparisons were conducted using independent samples t-test. For comparisons across different time points within each group, repeated measures analysis of variance was applied, followed by Bonferroni post-hoc tests. A significance level of *P* < 0.05 was used to determine statistical significance.

## Results

### Enrichment analysis reveals significant biological changes in the treatment process of DFU

DFU is a common complication that affects the quality of life and health of millions of diabetes patients worldwide. This chronic wound is not only difficult to heal but also prone to infections and, in severe cases, may result in amputation (Bolton 2022). Despite the availability of various treatments, such as pharmacotherapy, surgical interventions, and emerging biotechnological therapies, the effectiveness of treatment varies significantly among individual patients. This variability underscores the pressing need to identify reliable biomarkers to predict treatment responses and tailor personalized medical interventions (Agidigbi et al. 2023).

With the advancement of transcriptomics technologies, we are now able to systematically analyze the gene expression patterns of DFU patients to identify key genes associated with treatment response (Wang et al. 2021). However, traditional biological statistical methods often struggle to handle the high dimensionality and complexity of transcriptome data. In this context, machine learning techniques have shown immense potential in screening genes associated with treatment response due to their exceptional data mining capabilities and pattern recognition performance. By employing machine learning methods, we can not only effectively identify potential treatment response markers from large-scale transcriptome data but also gain a deeper understanding of the molecular mechanisms underlying DFU (Wang et al. 2022b).

**Fig. 1:**
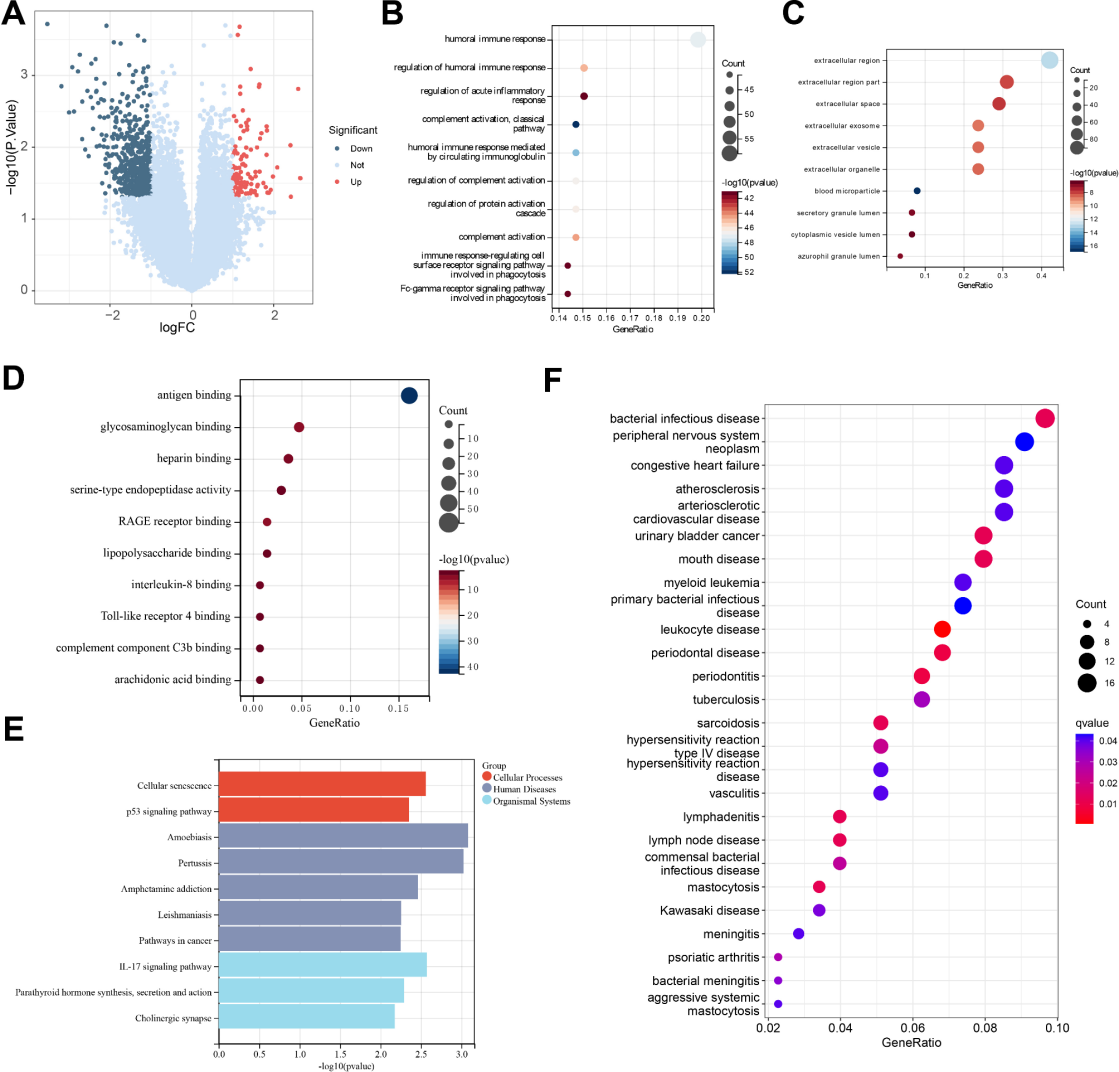
Enrichment analysis reveals significant biological changes during the dfu treatment process Note: (**A**) Volcano plot of DEGs in peripheral blood between pre-treatment and post-treatment healed DFU patients, with red indicating upregulated genes, blue indicating downregulated genes, and grey indicating genes with no significant difference; (**B**) Enriched BP in the GO enrichment analysis of DEGs; (**C**) Enriched CC in the GO enrichment analysis of DEGs; (**D**) Enriched MF in the GO enrichment analysis of DEGs; (**E**) Results of KEGG pathway enrichment analysis of DEGs; (**F**) Results of DO enrichment analysis of DEGs. The sample size for each group of patients was *n* = 15

To identify key genes associated with the treatment response of DFU and provide a scientific basis for personalized treatment of DFU, we downloaded a DFU-related transcriptome dataset from the GEO database and utilized machine learning techniques to assist in the analysis of the transcriptome data. By comparing the samples from the control group at week 0 after the start of treatment with the healed samples at 8 weeks post-treatment, we conducted an in-depth exploration. The differential analysis revealed that 102 genes were significantly upregulated in the experimental group, while 548 genes were significantly downregulated (Fig. 1A). Further, GO/KEGG enrichment analysis unveiled the biological expression differences, indicating that the DEGs were mainly enriched in BP such as humoral immune response, regulation of humoral immune response, and regulation of acute inflammatory response. The CC processes showed enrichment in the extracellular region, extracellular region part, and extracellular space. Moreover, in terms of MF, significant enrichment was observed in antigen binding, glycosaminoglycan binding, and heparin binding (Fig. 1B-D). Additionally, the KEGG enrichment analysis results suggested that the DEGs were mainly enriched in pathways such as Amoebiasis, IL-17 signaling pathway, and Cellular senescence (Fig. 1E). These results indicate that the treatment response of DFU may involve multiple BP and MF, particularly pathways associated with interactions related to immune response and extracellular regions. The significant enrichment of DEGs in immune regulation, especially in humoral immune response, regulation of acute inflammatory response, and extracellular region functions, suggests that these BP may play a crucial role in the healing process of DFU. Additionally, the enrichment of these genes in MF such as antigen binding and heparin binding implies their potential involvement in pathogen recognition and immune response, which is vital for infection control and wound healing (Rong et al. 2022; Theocharidis et al. 2020). KEGG pathway analysis further reveals the importance of the IL-17 signaling pathway and cellular senescence processes, indicating that the modulation of these pathways may be key mechanisms in treatment response. The IL-17 signaling pathway plays a central role in regulating inflammation and host defense, while the involvement of cellular senescence processes may impact cell turnover and wound repair efficiency (Zhang et al. 2022). The enrichment of the Amoebiasis pathway may suggest that specific infectious pathogens or related host responses could play a role in the pathological process of DFU.

Furthermore, we conducted a DO enrichment analysis to uncover the disease processes associated with the response to DFU treatment. The results revealed a strong association of numerous DEGs with certain diseases such as “bacterial infectious disease,” “leukocyte diseases,” and “hypersensitivity reaction.” These diseases demonstrated larger point sizes and lower q-values, indicating a high level of statistical significance in the enrichment (Fig. 1F). This enrichment pattern suggests that biological pathways related to these diseases may play a central role in the treatment response of DFU. Diseases like “congestive heart failure” and “atherosclerosis” were also identified in the analysis; despite involving fewer genes, their enrichment still exhibited significance, potentially reflecting a link between pathological changes in the cardiovascular system and the treatment response of DFU.

The above results indicate that DFU is closely associated with immune response, extracellular matrix interaction, and cardiovascular system changes. Furthermore, significant correlations were observed in specific disease pathways, such as bacterial infection and leukocyte diseases, providing important molecular targets and biological insights for future targeted treatment strategies.

### Machine learning for selection of DFU treatment response feature genes

**Fig. 2:**
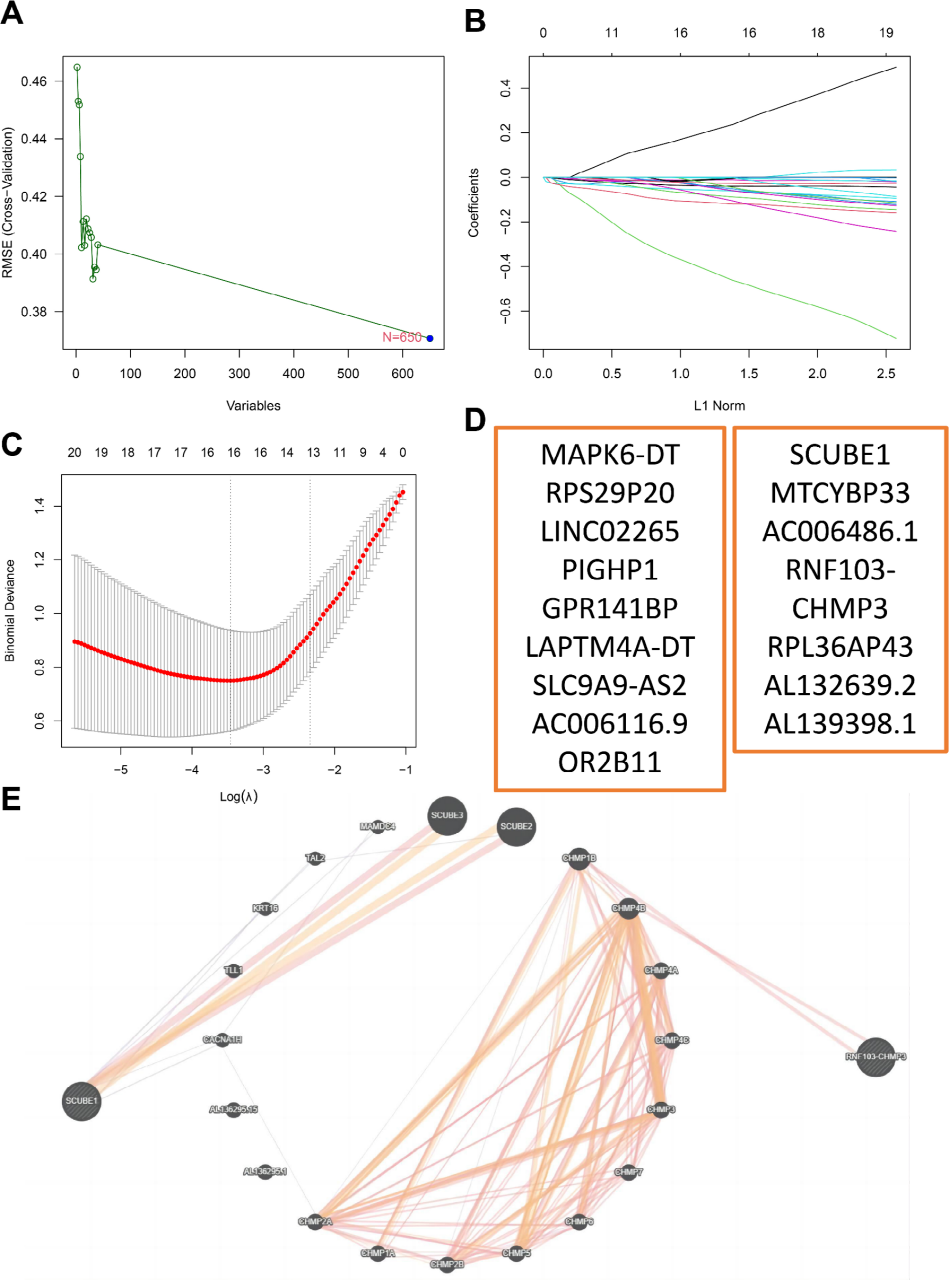
Machine learning algorithm for selecting feature factors in DFU treatment response Note: (**A**) SVM-RFE feature curve, with the x-axis representing the number of genes and the y-axis representing the cross-validation error; (**B**) Distribution of LASSO coefficients for DEGs; (**C**) Selection of the optimal parameter (lambda) for the LASSO model; (**D**) Feature factors selected for DFU treatment by machine learning algorithms; (**E**) Network of SCUBE1, RNF103-CHMP3, and their regulatory factors, where red lines indicate physical interactions, purple indicates co-expression relationships, and yellow suggests potential interactions

In order to further select characteristic genes for DFU treatment response, we utilized the Support Vector Machine-Recursive Feature Elimination (SVM-RFE) method to identify key gene expression features associated with the disease status. Based on the expression data of differentially expressed genes, we employed the rfe function for feature selection using cross-validation, incorporating SVM with radial basis kernel function and varying feature subset sizes. The analysis results were evaluated by plotting the relationship between the number of features and the RMSE of the model, indicating that all 650 differentially expressed genes met the optimal feature criteria (Fig. 2A). To further narrow down the number of feature genes, we conducted a regression analysis to fit the 650 differential genes and eliminate redundant features. The identified DFU candidate feature genes underwent Lasso regression using the “glmnet” function to model the data as a binary classification problem, with categories extracted from sample names as response variables. The model assessment involved plotting the model objects and applying cross-validation using “cv.glmnet” to determine the optimal lambda value (Fig. 2B). Ultimately, genes corresponding to non-zero coefficients extracted by the optimal lambda value were considered crucial genes related to the disease state, resulting in the final identification of 16 feature factors (Fig. 2C-D). Verification through the GeneCards online database eliminated long non-coding RNAs, pseudogenes, or other genetic elements, leading to the selection of feature genes SCUBE1 and RNF103-CHMP3. Furthermore, the prediction of their regulatory relationships and regulators was carried out using the GeneMANIA database, revealing a total of 23 regulatory factors (Fig. 2E).

Subsequently, we visualized the expression levels of SCUBE1 and RNF103-CHMP3 in the control and experimental groups, revealing significant downregulation post-treatment (Fig. S1A-B). Further investigation into their prognostic significance in DFU was conducted, plotting ROC curves which demonstrated that the AUC values of SCUBE1 and RNF103-CHMP3 were both greater than 0.8. This indicates their strong diagnostic value (Fig. S1C-D). These findings demonstrate that the machine learning algorithm successfully identified the DFU treatment response feature genes SCUBE1 and RNF103-CHMP3, which exhibit promising diagnostic potential.

To further test the diagnostic efficacy of SCUBE1 and RNF103-CHMP3, we validated their expression using the GSE80178 dataset. Our data showed that compared to non-foot ulcer samples (Non), the expression levels of SCUBE1 and RNF103-CHMP3 were elevated in diabetic foot ulcer (DFU) samples (Fig. S2A-B). Moreover, the ROC curve analysis revealed that the AUC values of SCUBE1 and RNF103-CHMP3 were both greater than 0.72, indicating a good diagnostic value (Fig. S2C-D).

### The immunoregulatory role of key feature factors

In previous analyses of BP, we observed that DFU is closely associated with immune responses, extracellular interactions, and cardiovascular changes. Moreover, they exhibit significant links in specific disease pathways, such as bacterial infections and leukocyte disorders. The immune system of diabetic patients often experiences functional impairment due to a hyperglycemic environment, affecting the functions of leukocytes, including chemotaxis, phagocytosis, and bactericidal activity, thereby rendering DFU susceptible to infections and delayed healing (Boulton et al., 2022; Ead and Armstrong 2023). Studies indicate that inflammatory cells, such as macrophages and neutrophils, play pivotal roles in the formation and healing of DFU, while the extracellular matrix provides a crucial scaffold for tissue reconstruction. Glycation under diabetic conditions and diabetes-related inflammation may disrupt normal matrix composition and extracellular signaling, thereby affecting wound healing processes (Zeng et al. 2022; Lin et al. 2022; Ead and Armstrong 2023).

The protein encoded by SCUBE1 is associated with angiogenesis and cell signaling, potentially influencing the function of vascular endothelial cells, thus playing a role in vascular changes and wound healing in diabetes (Lin et al. 2023). Proteins involved in the RNF103-CHMP3 complex may be implicated in endocytosis and the regulation of cell signaling, which could affect the release of inflammatory mediators and the immune response in wound healing (Wu and Lu 2019).

**Fig. 3:**
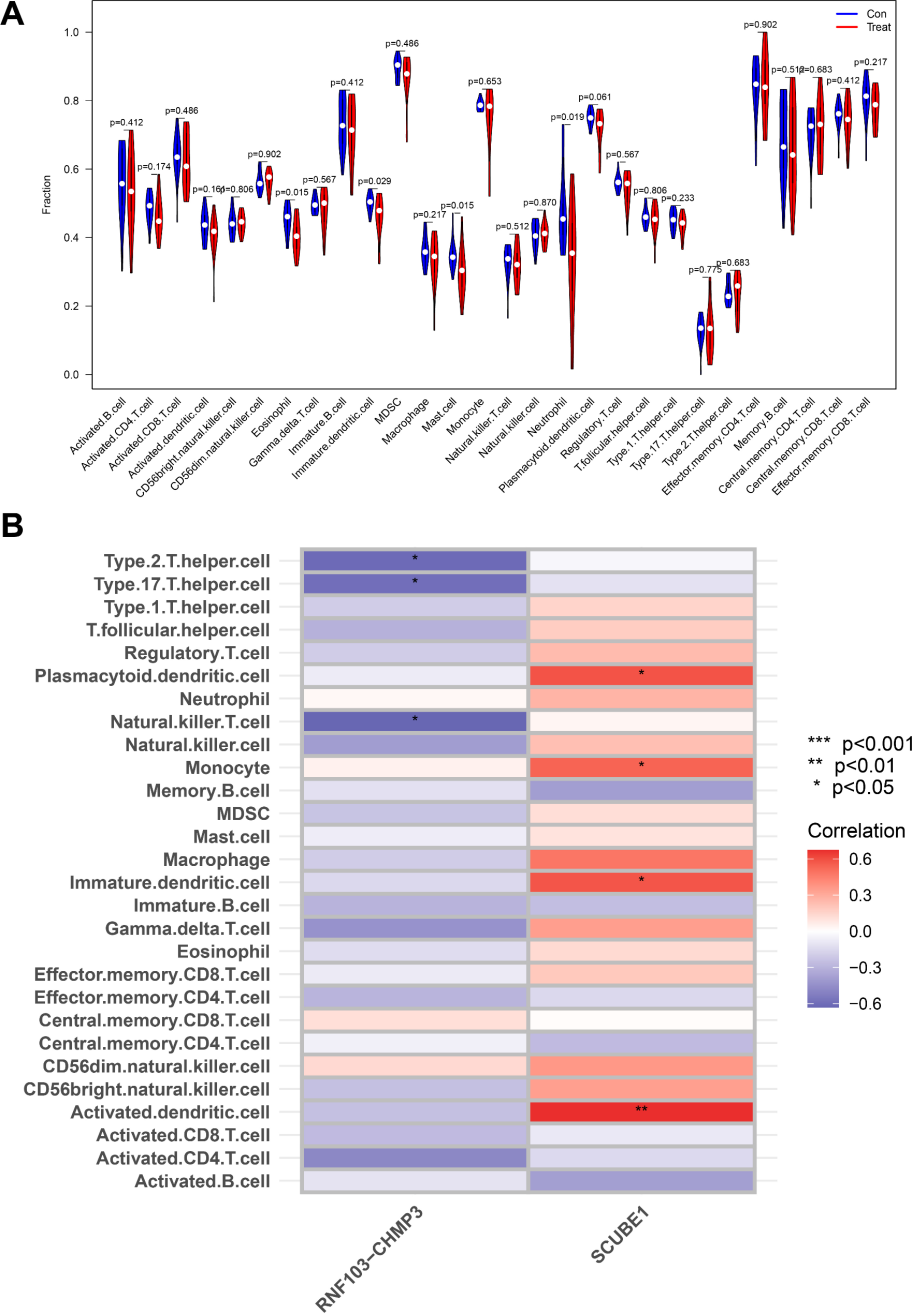
Immunomodulatory effects of key feature factors in the healing process of DFU Note: (**A**) Violin plots showing immune changes between the control group (DFU treated at 0 weeks post-infection) and the experimental group (DFU treated at 8 weeks post-infection, healed); (**B**) Heat map showing the correlation of SCUBE1 and RNF103-CHMP3 with immune cells. *** denotes *P* < 0.001 compared to the control group; ** denotes *P* < 0.01 compared to the control group; * denotes *P* < 0.05 compared to the control group

To further elucidate the impact of key regulatory factors on immune cells and activities in DFU, we conducted ssGSEA to investigate immune differences between the control group (treated 0 weeks post-DFU infection) and the experimental group (treated 8 weeks post-DFU infection, healed). The results revealed that compared to the control group, Eosinophils, Immature dendritic cells, Mast cells, and Neutrophils significantly decreased after treatment (Fig. 3A). Subsequently, we conducted an analysis of the correlation between feature genes and immune cells. The results revealed a significant negative correlation between RNF103-CHMP3 and Natural killer T cells, Type 17 T helper cells, and Type 2 T helper cells, while SCUBE1 showed a significant positive correlation with Plasmacytoid dendritic cells, Monocytes, Immature dendritic cells, and Activated dendritic cells (Fig. 3B). This outcome indicates that during the treatment of DFU, there were significant changes in the activity of specific immune cell populations, which were associated with the expression levels of key feature genes. The observed decrease in immune cells after treatment, such as Eosinophils, Immature dendritic cells, Mast cells, and Neutrophils, may reflect the positive impact of treatment in alleviating inflammation and promoting wound healing.

The gene RNF103-CHMP3 shows a significant negative correlation with Natural killer T cells, Th17 cells, and Th2 cells, indicating a potential inhibitory role of this gene in regulating the activity of these cell types. This may be in line with its low expression in diseased states, suggesting that during treatment and healing processes, related immune responses are weakened, contributing to a reduction in sustained inflammation. Additionally, the expression of SCUBE1 exhibits a significant positive correlation with Plasmacytoid dendritic cells, Monocytes, Immature dendritic cells, and Activated dendritic cells, suggesting that SCUBE1 may play an activating role in immune responses mediated by these cells. The downregulation of SCUBE1 after treatment may be associated with the adjustment of immune responses during treatment, aiding in reducing immune-mediated damage and promoting healing.

The results above indicate the presence of a complex interaction network between specific immune cells and key feature genes during the treatment and healing process of DFU. These genes are not only associated with expression changes during disease progression and specific immune cell activities, but their alterations may directly impact the regulation of inflammatory responses and the wound-healing process.

### Expression levels of SCUBE1 and RNF103-CHMP3 in single-cell transcriptome data

**Fig. 4:**
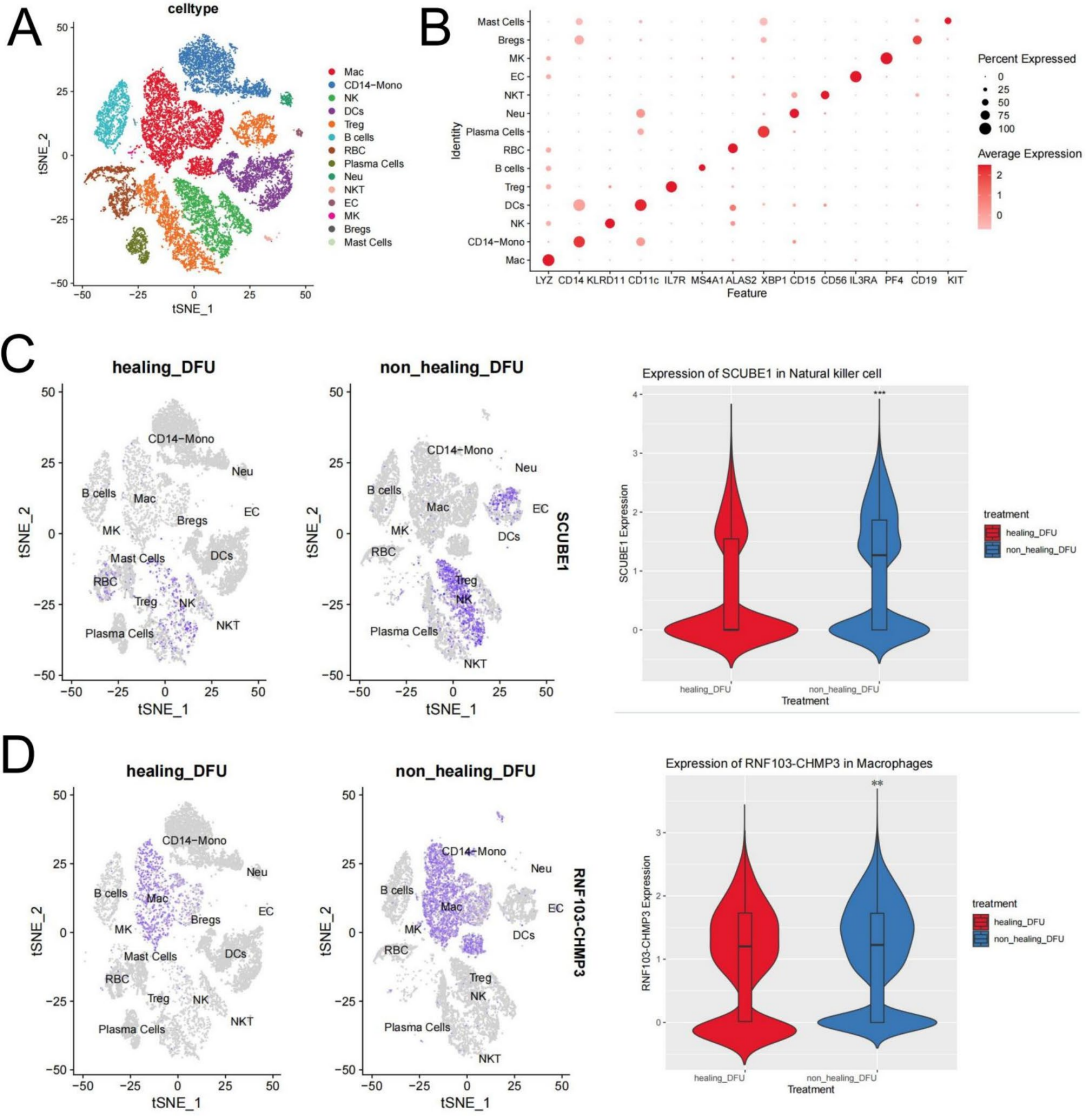
Single-cell RNA sequencing data showing different cell types and differential expression of key genes Note: (**A**) t-SNE clustering visualization shows the results of clustering single-cell RNA sequencing data using t-SNE, with different colors representing various cell types, including macrophages, NK cells, T cells, and others, totaling 14 cell types. (**B**) The dot plot shows the expression levels and percentages of selected genes across different cell types. The size and intensity of the color indicate the proportion of cells expressing the gene and the average expression level in the corresponding cell type. (**C-D**) Expression levels of SCUBE1 and RNF103-CHMP3 in single-cell transcriptome data, with the right panels showing the distribution of gene expression in different treatment groups (cured vs. uncured) in t-SNE space, where darker blue represents higher expression levels in those cells. *** indicates *P* < 0.001 compared to the control group; ** indicates *P* < 0.01 compared to the control group

PBMC samples from three subjects with cured DFU and two subjects with uncured DFU were downloaded from the GSE165816 dataset in the GEO database. The sequencing data were processed through normalization, scaling, clustering, and selection of highly variable genes. Dimensional reduction clusters are displayed in a 2D plot generated after t-SNE (PCA) clustering based on these 2000 highly variable genes (Fig. 4A). A total of 14 cell clusters were identified, and marker genes for each cell cluster are shown in Fig. 4B and Fig. S3. Further analysis revealed that SCUBE1 was primarily expressed in NK cells, with downregulated expression in NK cells of cured patients (*P* < 0.001) (Fig. 4C). RNF103-CHMP3 was expressed in macrophages, with reduced expression levels in macrophages of cured patients (*P* < 0.01) (Fig. 4D).

## Discussion

This study utilized machine learning techniques to identify genes associated with the treatment response of DFU, revealing SCUBE1 and RNF103-CHMP3 as potential molecular targets for early diagnosis and treatment of DFU. The identification of these genes not only elucidates the molecular mechanisms of DFU but also offers potential targets for future personalized treatment strategies. Our findings indicate significant changes in the expression of SCUBE1 and RNF103-CHMP3 in DFU patients, showing close associations with immune responses, extracellular interactions, and inflammation regulation. These discoveries are of paramount importance for understanding the pathogenesis of DFU and provide novel avenues for clinical interventions.

LASSO regression, implemented using the glmnet function, is widely used in biomedical research, particularly for identifying key genes associated with diseases due to its ability to handle high-dimensional data and reduce overfitting by selecting the most predictive features. The effectiveness of this approach has been validated in various studies. For example, one study employed LASSO regression to identify potential biomarkers in breast cancer, successfully extracting significant genes from large datasets (Liu and Wong 2019). Another study on cardiovascular diseases demonstrated that LASSO regression could identify important genes related to disease mechanisms and prognosis (Randhawa and Acharya 2015). Additionally, in liver disease research, LASSO regression was used to analyze gene expression profiles, identifying critical genes that serve as diagnostic markers (PMC8150479). Compared to previous studies, this research demonstrates significant methodological innovation (Chen et al. 2023; Huang et al. 2023; Schmidt et al. 2022). Traditional DFU studies have mainly relied on biomarker screening and functional validation, whereas this study uniquely integrates machine learning techniques with transcriptome data analysis, significantly enhancing the accuracy and reliability of gene selection (Li et al. 2023). We utilized LASSO regression models and SVM-RFE algorithms to screen feature genes associated with DFU treatment response from extensive gene expression data (Liang et al. 2024). This approach not only improves research efficiency but also reduces the impact of human bias, offering greater reproducibility and accuracy. Our study provides new perspectives and methods for exploring the molecular mechanisms of DFU.

SCUBE1 is a protein associated with the extracellular matrix and plays significant roles in biological processes such as angiogenesis, cell migration, and tissue regeneration (Lin et al. 2023). Studies have found that SCUBE1 levels are significantly elevated in the serum of diabetic patients, which may be associated with chronic inflammatory responses induced by diabetes. Inflammation is a crucial factor in the development of DFU, and SCUBE1 may contribute to DFU progression by modulating inflammatory responses (Synge 2013; Robin et al. 2017). The involvement of SCUBE1 in regulating immune responses suggests its critical role in DFU pathogenesis by influencing inflammation, which is essential for effective infection control and wound healing.

RNF103 (Ring Finger Protein 103) and CHMP3 (Charged Multivesicular Body Protein 3) are crucial regulatory factors in intracellular signal transduction and protein degradation processes. RNF103 has been shown to participate in inflammatory responses through the regulation of apoptosis pathways, while CHMP3 plays a vital role in multivesicular body (MVB) formation within cells. The formation of MVBs is closely related to intracellular signal transduction and material transport, processes that are critical for cellular communication and immune modulation in DFU (Isaacs 2018; Mathe et al. 2020). These pathways highlight how RNF103 and CHMP3 interact in DFU pathogenesis, emphasizing their roles in modulating inflammation and maintaining tissue integrity during wound healing.

The importance of the immune response in the pathogenesis of DFU has been widely recognized (Rubitschung et al. 2021; Senneville et al. 2024; Awasthi et al. 2022). The chronic inflammatory state of DFU leads to delayed wound healing and susceptibility to infections (Rodríguez-Rodríguez et al. 2021; Justynski et al. 2023). Our study demonstrates that SCUBE1 and RNF103-CHMP3 are closely associated with various immune cell activities, particularly in regulating inflammatory responses. This finding aligns with previous research indicating the dual role of the immune response in DFU, where it aids in infection resistance but may also lead to tissue damage due to excessive reactions (Liu et al. 2021a; Rubitschung et al. 2021; Senneville et al. 2024). By uncovering the roles of these key genes in immune regulation, we provide new evidence to enhance understanding of the immune mechanisms underlying DFU.

The extracellular interactions play a vital role in the pathological process of DFU (Liu et al. 2021b; Armstrong et al. 2021). Wound healing relies on the interactions between cells and the extracellular matrix, a process that is often severely disrupted in DFU patients (Chang and Nguyen 2021; Liu et al. 2022; Subramaniam et al. 2021). Our study reveals the significant involvement of SCUBE1 and RNF103-CHMP3 in extracellular interactions, aligning with previous research findings. Specifically, the role of SCUBE1 in cell migration and tissue remodeling may explain its importance in the wound healing of DFU. By elucidating the specific mechanisms of action of these genes, we offer a new perspective for the pathological research of DFU.

The potential applications of machine learning technology in the study of DFU are vast (Das et al. 2023; Cassidy et al. 2021; Haque et al. 2022). This study demonstrates that by integrating machine learning algorithms with transcriptome data analysis, not only has the accuracy of the research been enhanced, but it has also introduced new methods for investigating the molecular mechanisms of complex diseases. In the future, as data volume continues to increase and algorithms are further optimized, machine learning technology may play an increasingly important role in disease diagnosis, prognosis assessment, and personalized treatment. It is recommended that future research continues to explore the application of machine learning technology in the study of DFU and other complex diseases to further enhance the efficiency of gene selection and functional validation.

**Fig. 5:**
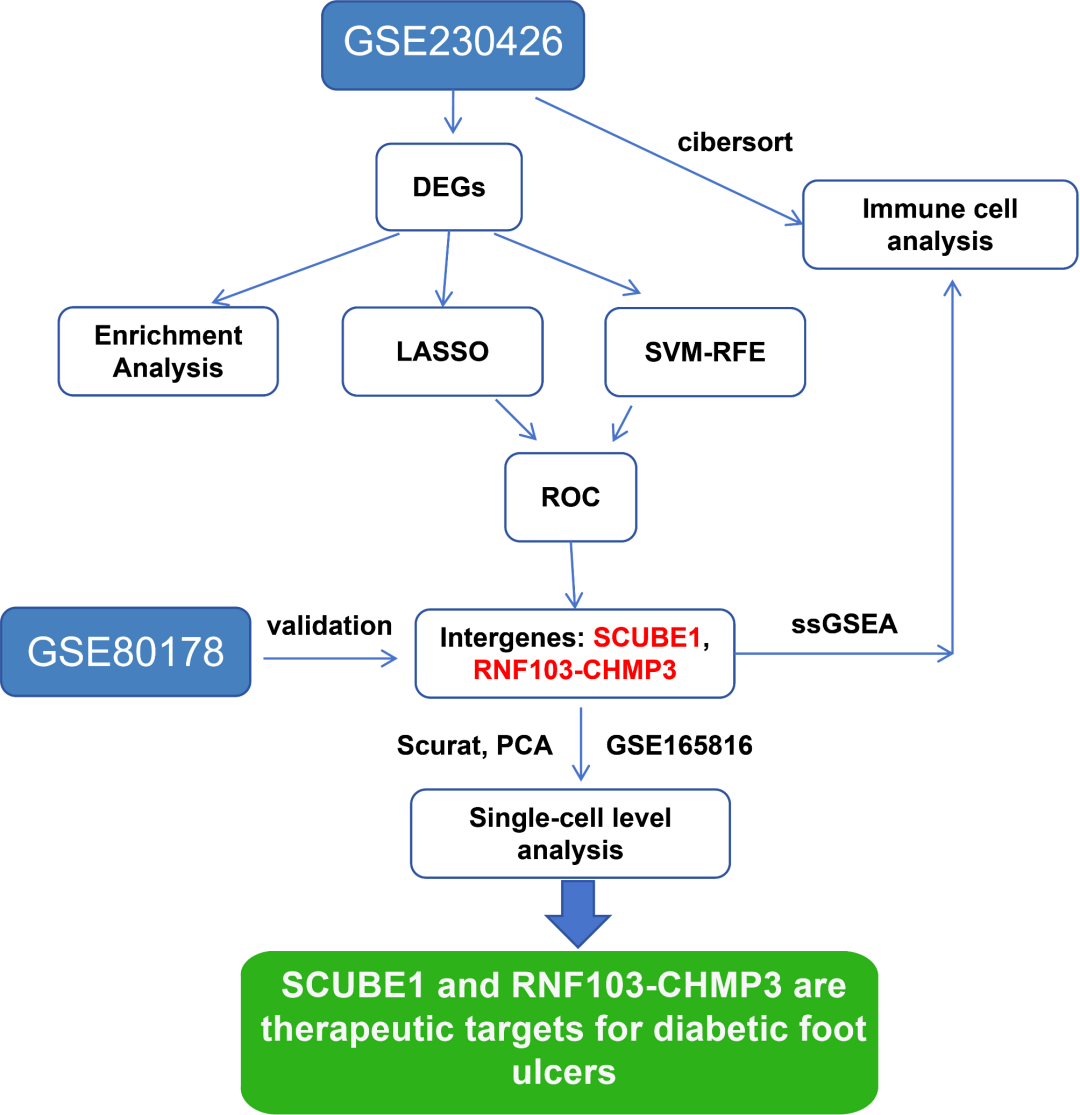
Bioinformatics Analysis Workflow for Molecular Screening of Treatment-Responsive Molecules in DFU

This study utilized machine learning techniques to analyze transcriptome data and successfully identified sensitive genes closely associated with the treatment response of DFU. Enrichment analysis was conducted to investigate the key BP enriched by these genes. Furthermore, a selection process was performed utilizing SVM-RFE and Lasso algorithms. By integrating immune correlation analysis, the study revealed the impact and mechanism of the candidate genes on DFU (Fig. 5). The results indicated a close association of DFU with immune response, extracellular interactions, and cardiovascular system changes. Moreover, significant correlations were observed in specific disease pathways, such as bacterial infections and leukocyte disorders. The machine learning algorithm identified candidate genes for DFU treatment response, including SCUBE1 and RNF103-CHMP3, which demonstrated good diagnostic value. These genes were closely associated with specific immune cell activities, and their variations could directly affect the regulation of inflammatory responses and wound-healing processes.

## Conclusion

This study employed machine learning techniques to identify the characteristic genes SCUBE1 and RNF103-CHMP3 in the treatment response of DFU from extensive transcriptome data. These genes play crucial roles in immune regulation and extracellular interactions in DFU, revealing the molecular mechanisms of DFU and providing novel biomarkers. The identification of SCUBE1 and RNF103-CHMP3 not only offers potential molecular targets for the early diagnosis of DFU but also lays the foundation for the development of personalized treatment strategies. These findings are expected to enhance the diagnostic accuracy and treatment outcomes for DFU patients, thus significantly improving their clinical prognosis and quality of life.

While this study has made significant progress in the field of DFU research, there are still some limitations that need to be acknowledged. Firstly, the transcriptome data used in the study were sourced from public databases, with a relatively small sample size, and technical variations and experimental conditions among different datasets may impact the accuracy of gene expression. Secondly, the selection and parameter settings of machine learning algorithms could influence the results of gene selection, necessitating further optimization and validation. Additionally, this study predominantly relied on bioinformatics analysis and machine learning techniques for data processing and gene selection, lacking experimental validation, which somewhat restricts the applicability and reliability of the research findings. Future efforts should focus on validating these discoveries through more clinical and experimental studies to ensure their effectiveness in practical applications.

Future research should focus on increasing sample sizes to enhance the universality and reliability of research results. Furthermore, optimizing machine learning algorithms and data processing methods can improve the accuracy and efficiency of gene selection. Combining bioinformatics analysis with experimental validation may aid in a more comprehensive understanding of the specific mechanisms of SCUBE1 and RNF103-CHMP3 in DFU and explore their potential roles in other diseases. The development of personalized treatment strategies based on these targets, such as specific drugs or gene therapy, holds promise for significantly improving the clinical prognosis of DFU patients. Through interdisciplinary collaboration and technological innovation, breakthroughs and advancements in the prevention, diagnosis, and treatment of DFU are anticipated, ultimately benefiting a larger number of patients.

## Electronic Supplementary Material

Below is the link to the electronic supplementary material.

Supplementary Material 1

## Data Availability

All data can be provided as needed.
