# Supplementary material for: Machine learning-driven discovery of novel therapeutic targets in diabetic foot ulcers
Source: Mol Med. 2024 Nov 14;30:215. doi: 10.1186/s10020-024-00955-z (PMC11562697; doi:10.1186/s10020-024-00955-z)
Supplement: Supplementary file 1 — Supplementary Material 1 [file 10020_2024_955_MOESM1_ESM.docx]

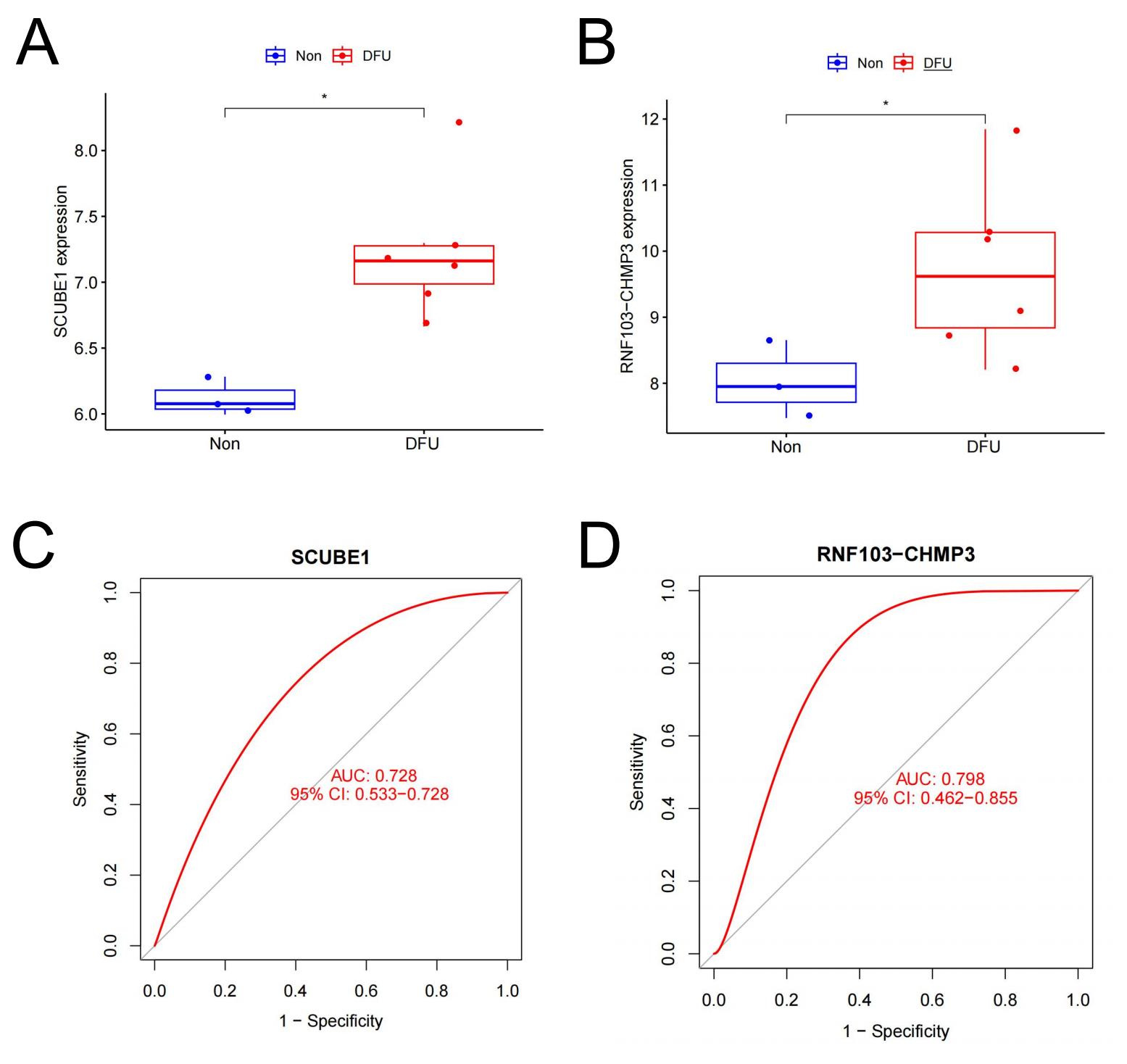


Fig. S1: Expression Profile of Feature Genes and ROC Curves

Note: (A) Expression profiles of SCUBE1 in the control and experimental groups; (B) Expression profiles of RNF103-CHMP3 in the control and experimental groups; (C) ROC curve for SCUBE1; (D) ROC curve for RNF103-CHMP3. *** indicates <Emphasis Type="Italic">P</Emphasis>&#x2009;&#x003C;&#x2009;0.001 compared to the control group


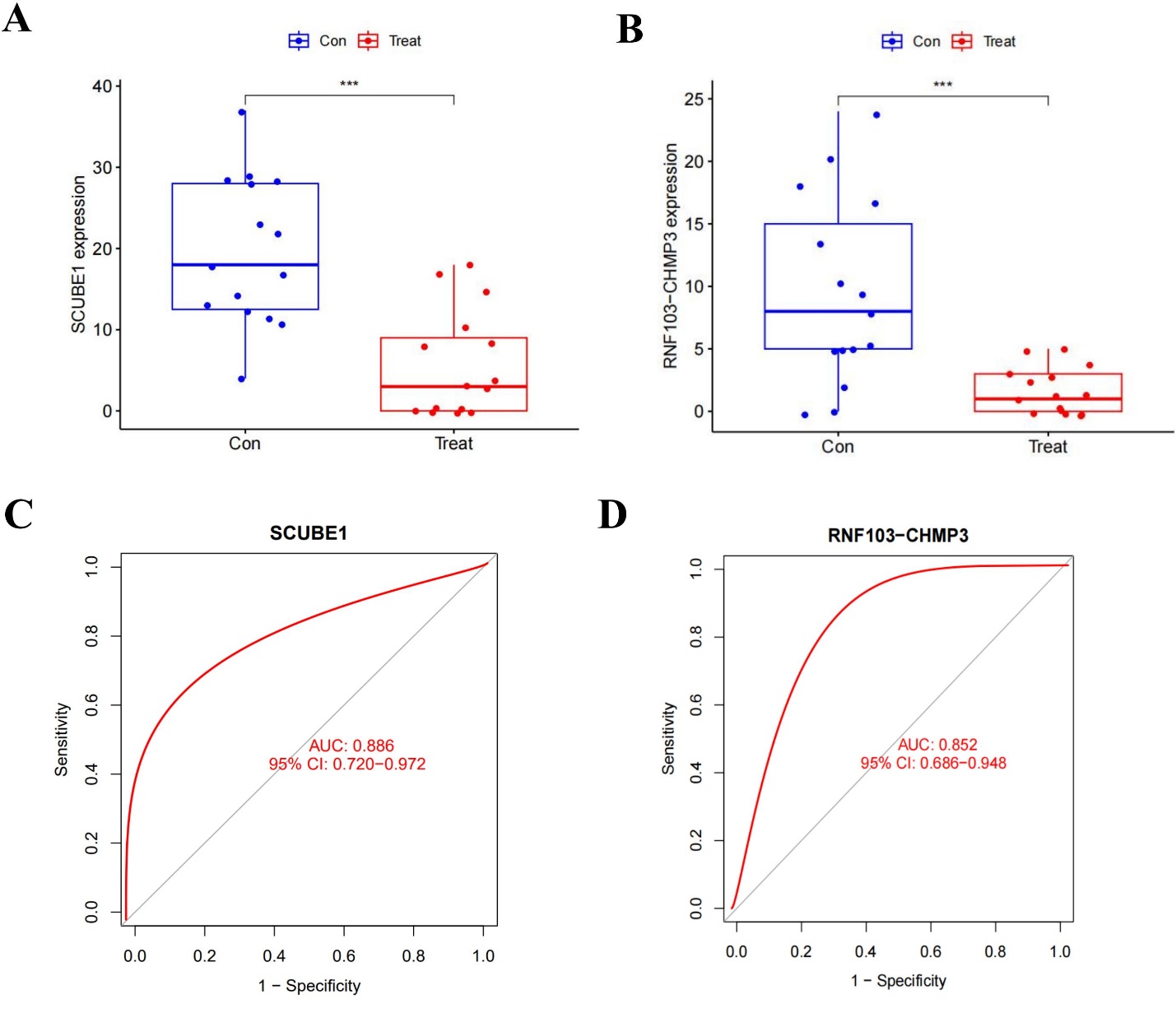


Fig. S2: Expression of Feature Genes and ROC Curves

Note: (A) Expression of SCUBE1 in non-foot ulcer (Non) and diabetic foot ulcer (DFU) groups; (B) Expression of RNF103-CHMP3 in non-foot ulcer (Non) and diabetic foot ulcer (DFU) groups; (C) ROC curve of SCUBE1; (D) ROC curve of RNF103-CHMP3. * indicates <Emphasis Type="Italic">P</Emphasis>&#x2009;&#x003C;&#x2009;0.05 compared to the control group


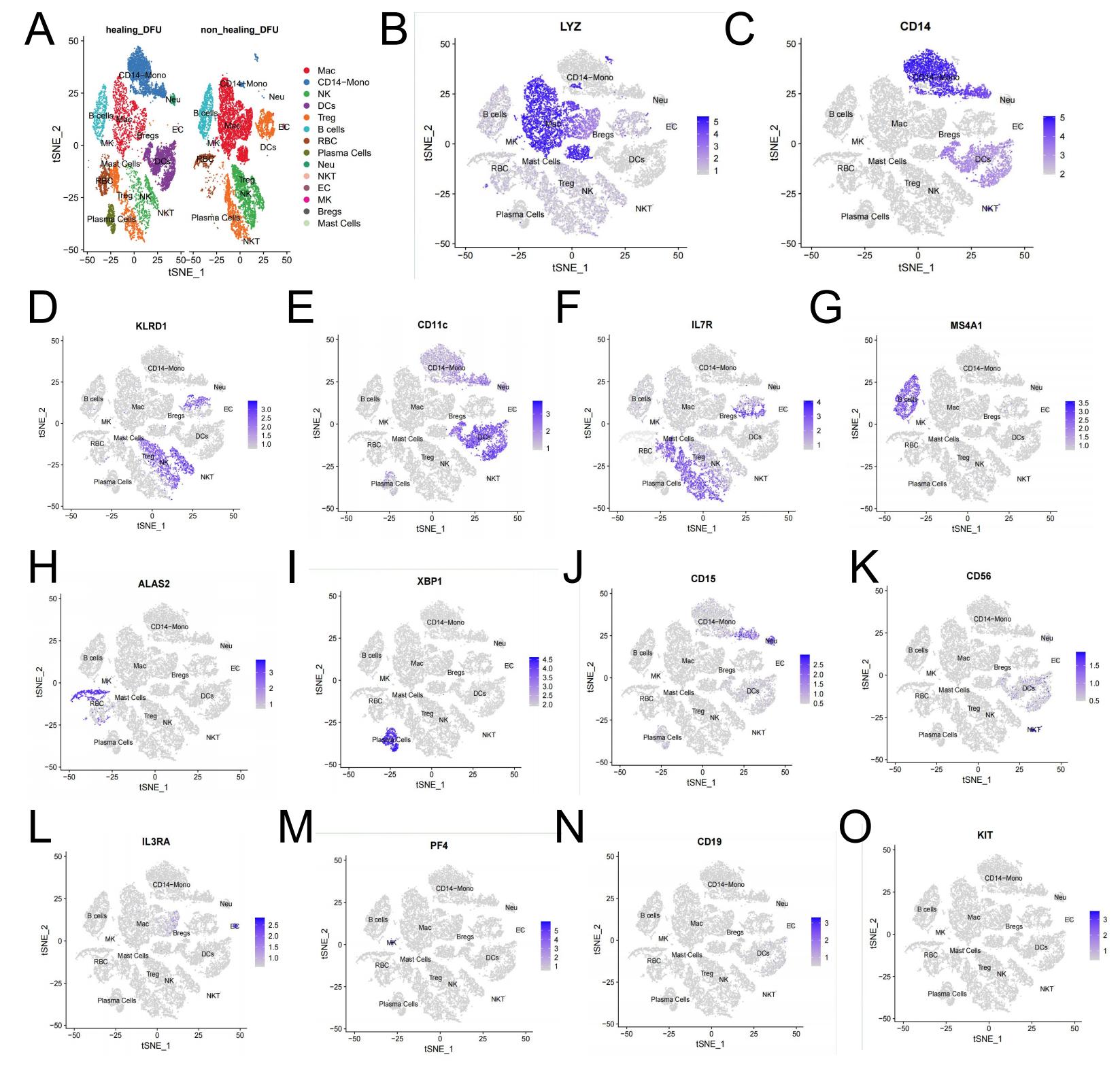


Fig. S3. Expression of Marker Genes in Various Cell Types in the Single-Cell RNA Sequencing Dataset.

Note: (A) t-SNE clustering distribution shows the distribution of different cell types analyzed using t-SNE technology, with color coding representing different cell types and the comparison between the two treatment conditions (healing_DFU and non_healing_DFU). (B-O) t-SNE plots display the expression levels of marker genes in different cell types, indicated by the intensity of blue.
